# Moderating factors in psilocybin-assisted treatment affecting mood and personality: A naturalistic, open-label investigation

**DOI:** 10.1007/s00213-024-06733-3

**Published:** 2025-01-07

**Authors:** Mona Irrmischer, Drew Puxty, Barış Onur Yıldırım, Jan Berend Deijen, Hessel Engelbregt

**Affiliations:** 1 GGZ Research, Hersencentrum Mental Health Institute, Amsterdam, The Netherlands; 2 PsyInternational Therapy, Utrecht, The Netherlands; 3 Novacare Solutions, Nijmegen, The Netherlands; 4 Faculty of Behavioural and Movement Sciences, Department of Clinical, Neuro- and Developmental Psychology, Section Clinical Neuropsychology, Vrije Universiteit, Amsterdam, The Netherlands; 5 GGZ Research, Academic Center for Trauma and Personality, Amsterdam, Netherlands; 6 Department of Psychiatry and Psychotherapy, Ludwig-Maximillians-University of Munich, Munich, Germany

**Keywords:** Psychedelic assisted therapy, Psilocybin, Depression, Anxiety, PTSD, Neuroticism, MEQ-30, PTGI, EBI

## Abstract

**Rationale:**

Psychedelic-assisted therapy is increasingly applied within mental health treatment.

**Objectives:**

This study focused on factors moderating changes in the acute and long-term effects of an individual psilocybin-assisted program on depression, anxiety, PTSD and personality structures by including demographic factors, subjective experience and degree of mystical type experiences during the dosing, as well as emotional breakthrough and personal growth after the program.

**Methods:**

At baseline, 1 week and 3 months after the psilocybin program participants completed the Generalized Anxiety Disorder Assessment (GAD-7), Patient Health Questionnaire (PHQ-9), PTSD Checklist for DSM-5 (PCL-5) and NEO Five-Factor Inventory-3 (NEO-FFI-3). In addition, after the dosing the Mystical Experiences Questionnaire (MEQ-30), Posttraumatic Growth Inventory (PTGI) and Emotional Breakthrough Inventory (EBI) were administered. Moderation effects were established using linear mixed-model analysis.

**Results:**

A single high dose of psilocybin in combination with therapy was found to lower symptoms of anxiety, depression, PTSD and neuroticism over a period of 3-months. Scores on openness and conscientiousness increased after the treatment only. Participants reported mystical type experiences, emotional breakthrough and personal growth. These subjective experiences together with demographic factors were moderating the observed positive changes.

**Conclusions:**

Findings indicate that individual psilocybin-assisted therapy has the potential for beneficial effects on mood and personality characteristics. Moreover, the study highlights the importance of subjective experiences and demographic factors in moderating this effect. This study adds to the ongoing research on psilocybin-assisted therapy by investigating contributing factors for optimizing this evolving type of therapy.

## Introduction

The emergence of psychedelic-assisted therapy marks a significant development in the field of mental health treatment (Luoma et al. 2020). This approach involves the supervised and controlled use of psychedelic substances, such as psilocybin (Griffiths et al. 2016), LSD (Krebs & Johansen, 2012), ketamine (Krupitsky and Grinenko 1997) and MDMA (Bouso et al. 2008; Wolfson et al. 2020) in combination with therapeutic support. A re-emergence of research into psychedelic substances as an adjunct to psychotherapy has shown promising results for PTSD, anxiety and depression (Mitchell et al. 2021; Vargas et al. 2020), with a recent review showing effects larger than typically found in trials of psychopharmacological or psychotherapy interventions (Luoma et al. 2020).

For psilocybin robust reductions in depressive and anxiety symptoms have been demonstrated following one or two doses (Carhart-Harris et al. 2016; Davis et al. 2021; Griffiths et al. 2016; Grob et al. 2011; Ross et al. 2016). Of note, positive responses to psilocybin treatment with psychological support were maintained at a 6-month follow-up, well beyond the acute pharmacological actions of the psilocybin itself (Carhart-Harris et al. 2018). More recently, a trial comparing psilocybin with escitalopram, a SSRI antidepressant, in patients with long-standing, mild-to-severe depression demonstrated a similar reduction in depressive complaints compared to baseline at a 6-week follow-up (Carhart-Harris et al. 2021).

Research exploring psilocybin therapy on personality structures in volunteers with treatment-resistant depression demonstrated reduced neuroticism, alongside increased openness and extraversion at a 3-month follow-up (Erritzoe et al. 2018). Studies in healthy volunteers revealed increases in the domain of openness that endured at a 1-year follow-up in participants who reported a mystical-type experience (MacLean et al. 2011). Moreover, data obtained from an online survey demonstrated lifetime psychedelic use to be predictive of increased openness (Nour et al., 2017). In contrast, no changes in openness were reported following administration of LSD in healthy volunteers (Schmid and Liechti 2018).Taken together, psychedelic use appears to be associated with personality changes, although no definitive conclusions can be made.

The rationale underlying the use of psychedelics as an adjunct to psychotherapy is broad. First, the acute pharmacological action appears to promote increased top-down regulation of limbic structures (Calder and Hasler 2023), thereby reducing fear and arousal, leading to enhanced emotional and cognitive processing of traumatic material. Second, psychedelics may serve to strengthen the therapeutic relationship through fostering enhanced trust and rapport; or through facilitating the process of fear extinction (Catlow et al. 2013; Krediet et al. 2020). Third, psychedelics have been shown to promote neural plasticity (neurogenesis, spinogenesis, synaptogenesis) in vivo and in vitro (Ly et al. 2018). It is postulated that psychedelic-induced neural plasticity could mediate the rapid antidepressant and anxiolytic effects observed within clinical trials.

Interest is also growing regarding extra-pharmacological factors underlying the enduring therapeutic effects of psychedelic assisted therapy with regards to treatment protocols and subjective experience. There appears to be a relationship between therapeutic outcomes and ratings of subjective experience (e.g., music, peak experience) during a psychedelic dosing session (Garcia-Romeu et al. 2015; Griffiths et al. 2016; Kaelen et al. 2018; Majić et al. 2015; Roseman et al. 2018). Moreover, the importance of ‘set’ and ‘setting’ and the general context concerning psychedelic use has also been shown to influence observed outcomes. Clinical trials documenting positive outcomes place a high emphasis on the ‘set’ of each participant through detailed screening procedures and extensive preparation therapy. At the same time, use of a carefully curated therapeutic ‘setting’, characterized by a high degree of care from trained mental health professionals to provide safety and to facilitate conditions for the participant to be able surrender to the experience (Carhart-Harris et al. 2018). Conversely, less favorable results have been found when the importance of ‘set’ and ‘setting’ has been neglected (Oram 2014).

In addition, data from a prospective study highlights the importance of individual experiences during the psychedelic session. Specifically, higher ratings of a ‘mystical-type experience’ (as measured by the MEQ-30) were predictive of positive changes in wellbeing, whilst having a clear ‘intention’ was found to be conductive for a mystical-type experience (Haijen et al. 2018). The MEQ-30 (30-item revised Mystical Experiences Questionnaire) is the most recently developed version of the MEQ, which contains four factors: mystical, positive mood, transcendence of space and time, and ineffability (MacLean et al. 2012).

Other acute effects that appear to play a moderating role in the enduring positive outcomes include experiences of ‘emotional breakthrough’. The experience of an emotional breakthrough has been posited to overlap with the psychoanalytic term of ‘catharsis’ and is thought to be an additional mediator of the long-term responses to psychedelics. Investigators utilizing an online prospective study (N = 379) constructed and validated the ‘Emotional Breakthrough Inventory’ (EBI) in predicting changes in well-being following a psychedelic experience (Roseman et al. 2019). Similarly, reduced experiential avoidance (avoidance of emotion) as well as increased connectedness and acceptance of emotional experience appear to have a moderating effect on the observed positive outcomes (Watts et al. 2017).

Acknowledging the importance of integration after the experience, this is the first study implementing the post traumatic growth questionnaire (PTGI) in a psilocybin setting. The 21-item scale was developed as an instrument to investigate growth and self-improvement after traumatic events, highlighting transformation resulting from the extreme experience. It includes the factors: ‘Seeing new Possibilities’, ‘Relating to Others’, ‘Personal Strength’, ‘Spiritual Change’, and ‘Appreciation of Life’ (Tedeschi and Calhoun 1996).

The main objective of the present study is to examine factors that were moderating changes in the acute and long-term effects of an individual psilocybin-assisted program on depression, anxiety, PTSD and personality structures by including demographic factors, subjective experience and degree of mystical type experiences during the dosing, as well as emotional breakthrough and personal growth after the program.

## Methods

### Participants

This study includes data from 83 participants (46 females, 55.4%) who all underwent an individual psilocybin-assisted treatment program with a standardized protocol. Participants were not preselected or recruited for any health concerns rather they enrolled in the program out of own incentive through word of mouth and internet search. They had a mean age of 42.3 years (*SD*: 2.4 years), were predominantly highly educated (university degree, *n* = 65, 78%) and in employment at the onset of the study (*n* = 76, 92%). Testing against a reference population indicated that participants in the present study experienced higher baseline depression, anxiety, PTSD and neuroticism. Nearly half (*n* = 37, 45%) had a prior or current psychiatric diagnosis, including depression, anxiety or PTSD, and a third had been prescribed psychiatric medication at some point in their life (*n* = 30, 36%). These psychological complaints were often acute (in the last 1–2 years, 28%) or long-term complaints (10 + years, 33%). The average alcohol consumption was low to moderate (1–2 drinks per week, *n* = 35, 42%, 3–7 drinks per week, *n* = 28, 34%), and most (*n* = 74, 89%) had never experienced a high dose of psilocybin. The participants had tried on average 6 other alternative healing modalities (e.g., mindfulness, Yoga, acupuncture) before entering the study. See Table 1 for demographic information.

**Table 1:** Demographic data of participants entering the program

Total		*N* = 83(%) ± SD
Gender	Female	34 (41)
	Male	46 (55.4)
	Missing/no response	3 (3.6)
Age	In years	42.3 ± 12.7)
	Missing/no response	3 (3.6)
Education	High School diploma	9 (10.8)
	University (or equivalent)	65 (78.3)
	Other	4 (4.8)
	Missing/no response	5 (6)
Employment status	Working	76 (91.6)
	Unemployed	2 (2.4)
	Missing/no response	5 (6)
Psychiatric history	Has been diagnosed with at least one psychiatric illness	37 (44.6)
	Never been diagnosed	41 (49.4)
	Missing/no response	5 (6)
Medication	Yes	30 (36)
	No	48 (57.8)
	Missing/no response	5 (6)
Alcohol consumptions (drinks per week)	0	8 (9.6)
	1–2	35 (42.2)
	3–7 7+	28 (33.7) 6 (7.2)
	Missing/no response	6 (7.2)
Prior experience with psychedelics	Never (Psychedelic naive)	45 (54.2)
	Microdose	29 (34.9)
	At least one Macrodose	4 (4.8)
	Missing/no response	5 (6)
Support system	None	10 (12)
	Family only	11 (13.3)
	Professional (doctor/psychiatrist/psychologist) only	26 (31.3)
	Professional and family	31 (37.3)
	Missing/no response	5 (6)
Alternative healing modalities	Total number of alternative modalities tried	6 (± 3.5)
	Missing/no response	5 (6)
Aware of abuse	No	45 (54.2)
	Yes, during childhood	23(27.7)
	Yes, during adolescence	8 (9.6)
	Yes, during adulthood	2 (2.4)
	Missing/no response	5 (6)
How long having psychological complaints (years)	0	8 (9.6)
	1–2	23 (27.7)
	2–4	10 (12)
	5–9	10 (12.0)
	10+	27(32.5)
	No response	5 (6)

Absolute frequencies and means ± standard deviations are presented in the table. Numbers in the brackets show the percentage of absolute frequencies

### Study procedure

The individual psilocybin-assisted treatment program consisted of an intake phase, preparation, dosing session, and integration.

#### Intake

Each participant first completed the general intake and baseline complaints questionnaires (see *Study Variables*). These were used for screening purposes and evaluated by a psychiatrist and a psychologist together with a set of inclusion and exclusion criteria (See Appendix 1 for full inclusion / exclusion criteria).

#### Preparation

Upon admission to the program each participant underwent a 1 h online and 2 h in-person preparatory therapy as well as an additional 1 h in-person session for practical preparation (e.g., instructions for navigating the experience, establishing safety) with a facilitator.

#### Dosing session

Each participant was provided with an amount of fresh truffle sclerotia (see Appendix 3) equivalent to 25 mg psilocybin. The facilitator stayed with the participant throughout the whole day.

#### Integration

Follow-up therapy took place for 2 h in person the day after the dosing and 1 h online 1 week later. In total each participant received a combined total of 6 h with a psychotherapist, and ca. 9 h with a facilitator (for more details see Appendix 2).

### Study variables and questionnaire administration

The questionnaires at the intake phase included a general health and demographics intake questionnaire and the baseline measures of the GAD-7, PHQ-9 and PCL-5. These were mandatory for admission and sent out by the care coordinator online and filled in by all participants.

All additional questionnaires (post dosing and 3-month follow-up GAD-7, PHQ-9 and PCL-5, the NEO-FFI-3, MEQ-30, PTGI and EBI) were sent out online by the therapists. These were not mandatory for participating in the program anymore but voluntary for research purposes. This construct resulted in failures to send out the questionnaires by some therapists. Missing data therefore was random due to either participants not receiving the questionnaire and non-responders due to lack of enthusiasm or incentive to participate in research.

**Fig. 1:**
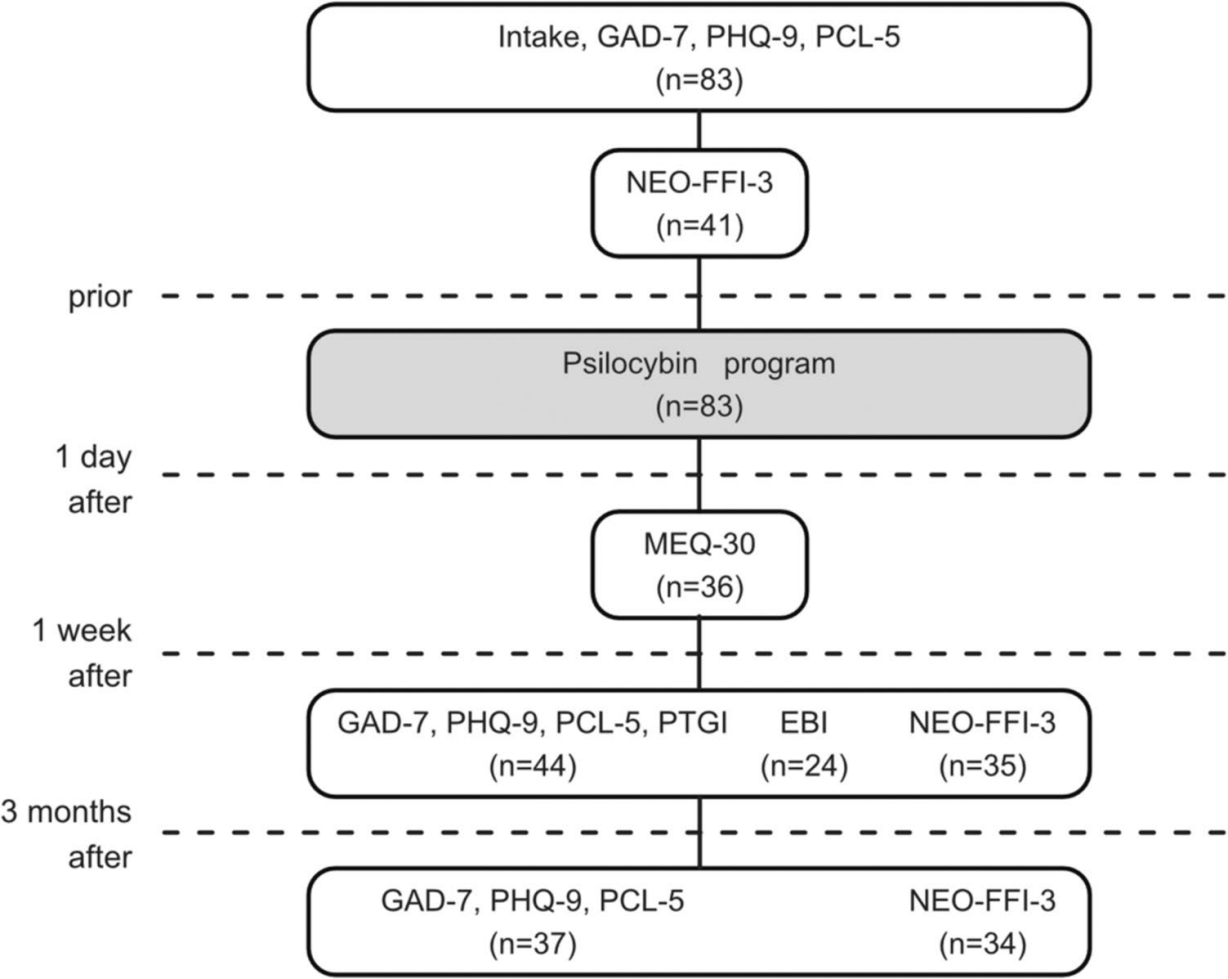
Flow chart. administration of questionnaires

The baseline GAD-7, PHQ-9, PCL-5 questionnaires were filled out at the intake by 83 participants, of which only the data of the participants that also filled in the post dosing questionnaires were used for the following GAD-7, PHQ-9, PCL-5 analysis, which included 44 participants post dosing and 37 at follow-up. The baseline NEO-FFI-3 was filled out after the intake by 41 participants, at post dosing by 35 and at follow-up by 34. 1 day after the dosing the MEQ-30 was filled out by 36 participants. One week after the dosing the PTGI was filled out by 44 participants. The EBI was added later and filled out by 23 participants, in order to include emotional breakthrough, but it was only analyzed as treatment outcome and not included in the moderation analysis due to late addition and too little cases for the moderation analysis (see Fig. 1 flow chart).

As stated above the missing data is of rather random nature, which makes the mixed model approach using maximum likelihood estimations work robustly (Enders 2022).

Further would even a possible response bias not negatively impact the main objective of this study as it is not aimed at proving the clinical efficacy of the treatment, which has been demonstrated elsewhere (Carhart-Harris et al. 2016; Davis et al. 2021; Griffiths et al. 2016; Grob et al. 2011; Ross et al. 2016), but is aimed at investigating the moderating factors that facilitated the positive change observed.

### Short description of questionnaires

#### Intake and Demographics Questionnaire

In-house survey for collecting information on general health, demographics and intentions.

#### Generalized Anxiety Disorder 7 (GAD-7) (Primary)

7-item used as an initial screening tool for generalized anxiety disorder (Spitzer et al. 2006).

#### Patient Health Questionnaire-9 (PHQ-9) (Primary)

9-item used for screening, diagnosing, monitoring and measuring the severity of depressive complaints (Kroenke et al. 2001).

#### The PTSD Checklist for DSM-5 (PCL-5) (Primary)

20-item that assesses the presence and severity of PTSD symptoms. (Cernovsky et al. 2021).

#### NEO-FFI-3 Five-Factor Inventory-3 (FFI-3) (Primary)

60-item version measures the five domains of personality: neuroticism, extraversion, openness, agreeableness, and conscientiousness. (McCrae and Costa 2007).

#### The Mystical Experiences Questionnaire (MEQ-30) (Secondary)

this 30-item version measures the quality of a mystical experience. The four factors are mystical, positive mood, transcendence of space and time, and ineffability (Barrett et al. 2015).

#### Posttraumatic Growth Inventory (PTGI) (Secondary)

21-item scale for evaluation of personal growth that follows a stressful experience. (Tedeschi and Calhoun 1996).

#### Emotional Breakthrough Inventory (EBI) (Secondary)

6-item measuring emotional breakthrough following a psychedelic experience on a 0 to 100 scale. (Roseman et al. 2019).

#### Experience Description

In-house survey to measure surrender and personal significance on a 0 to 100 scale and collection of personal experience descriptions.

### Statistical analysis

First, to ascertain that the smallest measurement (34 participants) provided enough power to detect the hypothesized effects, we conducted a power analysis for repeated measures ANOVA using the program G*Power 3.1.9.4 (Faul et al. 2007). After applying a medium effect size η^2^ = 0.13 (similar to *f* = 0.25), correlation = 0.5 and 3 measurements, the obtained power was 0.81 for a sample size of 28.

Baseline analyses of the GAD-7, PHQ-9, PCL-5 and the NEO-FFI-3 facets (neuroticism, extraversion, openness, agreeableness and conscientiousness) were performed by adding the total score and then comparing them with representative samples of the general population using between group t-tests.

To investigate the outcome of the treatment on the MEQ-30, PTGI & EBI the average scores for each were calculated by adding the total scores and comparing them to other treatment outcomes from literature, using between group t-tests. For the MEQ-30 the total scores were also calculated as % of total (h score/30; positive mood score /30; ineffability score/15; mystical score /75; total score/150). Additionally, single word qualitative descriptions of the experience were collected which will be reported in % averages.

To examine the long-term effects of the treatment on the outcome of the GAD-7, PHQ-9, PCL-5 and NEO-FFI-3 facets, the average scores were calculated and analyzed for the three time points (before dosing session, after dosing session, follow-up) using a basic mixed-effects linear model analysis for each with time included as a factor.

To explore possible moderating factors on the treatment outcome over time, each of the basic models were extended with variables available from the intake and additional questionnaires (see Table 1 & *description of questionnaires*). In this exploratory analysis, first the influence of demographic moderators (gender, age, alcohol intake, diagnosis, ‘years of having complaints’, reported abuse, support system and ‘prior experience with psilocybin’) present *before* the session were tested. Then measures of subjective experience (‘Ability to Surrender’, ‘Emotional experience’ and the 4 facets of the MEQ-30) *during* the experience, and last changes *after* the experience (5 facets of the PTGI). The reasoning behind testing multiple models (before, during and after) lies in feasibility of applying the available data in a comprehensive and meaningful way without overloading the model with too many variables. The reasoning behind testing each facet of the MEQ-30 and PTGI lies in the exploratory nature of this study, to gain more nuanced insight.

The models were tested for main effects of the moderators and their interaction effects with time. For openness and conscientiousness only main effects, and no interaction with time, were included due to the lack of significance of time in the basic model (for the mixed-effects linear model formula see section *supplemental material*).

The model fit was tested using the Akaike information criterion (AIC) (Akaike 1974). Results were tested at a significance level of *p* < 0.05 and corrected for multiple comparisons using the Benjamini Hochberg false discovery rate (FDR, 0.05) correction method (Benjamini and Hochberg 1995). Data was analyzed using IBM SPSS Statistics (version 28) (IBM 2021).

## Results

### Baseline analysis PHQ-9, PCL-5, GAD-7 and NEO-FFI-3

Average scores of the **PHQ-9** baseline were indicative of mild depression (Kroenke et al. 2001) and testing against a reference population (age 48.9 ± 18.1, 53.6%women, *n* = 5013; Kocalevent et al. 2013) indeed showed that participants in the present study experienced higher baseline depression (Msample = 4.52 ± 3.99, Mpopulation = 2.91 ± 3.52, *p* = 0.0003).

Average baseline scores on the **PCL-5** indicated the presence of some PTSD symptoms (Cernovsky et al. 2021) and testing against a reference population (age 52 (13.5) years, 68% women, *n* = 126; Tu et al. 2021) showed that participants in the present study experienced higher baseline PTSD (Msample = 12.43 ± 11.37, Mpopulation = 5.8 ± 6.9, *p* < 0.0001).

Average scores of the **GAD-7** baseline were indicative of mild anxiety (Spitzer et al. 2006) and testing against a reference population (age 48.4 ± 18.0, 53.6% women, *n* = 5030 ; Löwe et al. 2008) indeed showed that participants in the present study experienced higher baseline anxiety (Msample = 4.62 ± 3.65, Mpopulation = 2.95 ± 3.41, *p* = 0.0001).

Testing the **NEO-FFI-3** against a reference population (56% female, ages 21–91) that was highly educated and predominately Caucasian, (*n* = 635; McCrae and Costa 2007) indicated that participants in the present study had significantly higher neuroticism (Msample = 27.05 ± 8.97),Mpopulation = 20.8 ± 7.7,*p* = 0.0001),extraversion(Msample = 30.59 ± 8.86, Mpopulation = 28.2 ± 6.2, *p* = 0.034) and openness (Msample = 37.6 ± 7.55, Mpopulation = 28.4 ± 6.3, *p* = 0.001). Mean scores on agreeableness and conscientiousness were not significantly different.

### Treatment effects over time: PHQ-9, PCL-5, GAD-7and NEO-FFI-3

To test if the intervention had an effect on psychological complaints after the dosing and also at the 3-month follow-up, a basic mixed-model analysis was performed with time included as a factor.

#### PHQ-9

**Fig. 2:**
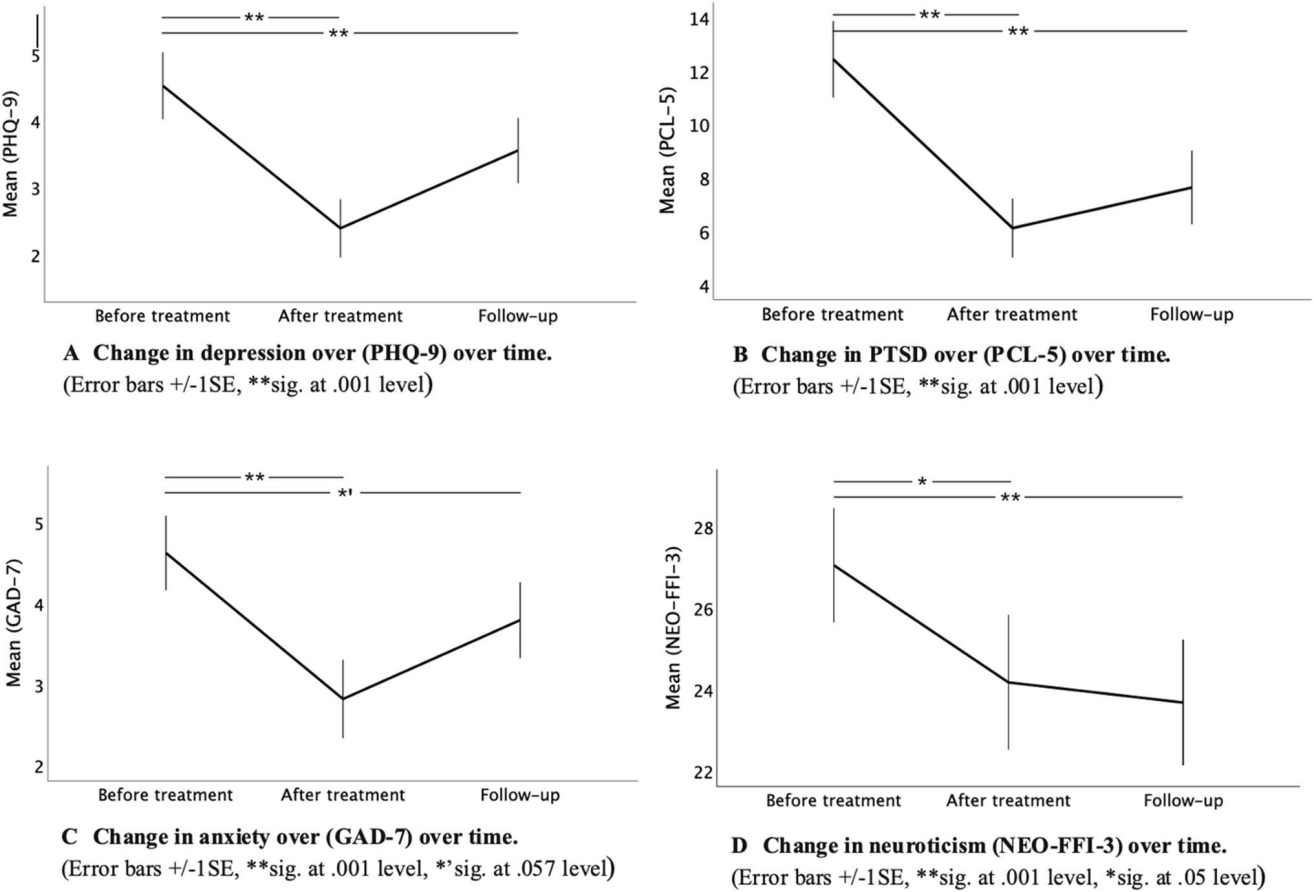
Change in depression, PTSD, anxiety and neuroticism. Measurement points are before the program, 1 week after and 3-month follow-up

The effect on depression was significant over time (*F*(2, 42.4) = 8.58, *p* < 0.001). The complaints reduced from 4.52 ± 4 at baseline to 2.39 ± 2.9 after the dosing (*p* < 0.001). Although the average score increased again to 3.55 ± 3.0 at the 3-month follow-up, which was significantly higher (*p* = 0.019) than after dosing, it was still significantly lower than at baseline (*p* = 0.001) (Fig. 2a).

#### PCL-5

The effect on PTSD was significant over time (*F*(2, 46.52) = 13.26, *p* < 0.001). The complaints reduced from 12.43 ± 11.4 at baseline to 6.11 ± 7.3 after the dosing (*p* = < 0.001). Although the average score increased again to 7.63 ± 8.5 at the 3-month follow-up, it was not significantly different from right after the dosing, but it was still significantly lower than baseline (*p* < 0.001) (Fig. 2b).

#### GAD-7

Anxiety showed a significant effect over time (*F*(2, 55.61) = 6.21, *p* = 0.004). The complaints reduced from 4.62 ± 3.6 at baseline to 2.82 ± 3.2 after the dosing (*p* = 0.001). Average score increased again to 3.79 ± 2.9 at the 3-month follow-up, it was not significantly different from right after the dosing but was still on the threshold of being significantly lower than baseline (*p* = 0.057) (Fig. 2c).

Similarly, to test if the intervention had an effect on personality (NEO-FFI-3) after the dosing and also at the 3-month follow-up, a basic mixed-model analysis was performed with only time included as a factor.

#### Neuroticism

Showed a significant effect over time (*F*(2, 34.7) = 9.16, *p* < 0.001). neuroticism reduced from 27.05 ± 9.0 at baseline to 24.17 ± 9.8 after the dosing to 23.68 ± 9.0 at the 3-month follow-up. The change from baseline to after the dosing as well as at follow-up was significant (*p* = 0.003 and *p* = 0.001, respectively) (Fig. 2d).

The effect on **openness** and **conscientiousness** did not show a significant interaction with time, only a significant increase from baseline (37.61 ± 7.6 and 33.2 ± 7.6) to after the dosing (38.11 ± 8.8 and 34.4 ± 7.4) was seen in both (*p* = 0.028 and *p* = 0.018, respectively).

NEO-FFI-3 extraversion and agreeableness showed no significant effect.

### Additional treatment outcomes: MEQ-30, PTGI, EBI & experience

#### MEQ-30

**Fig. 3:**
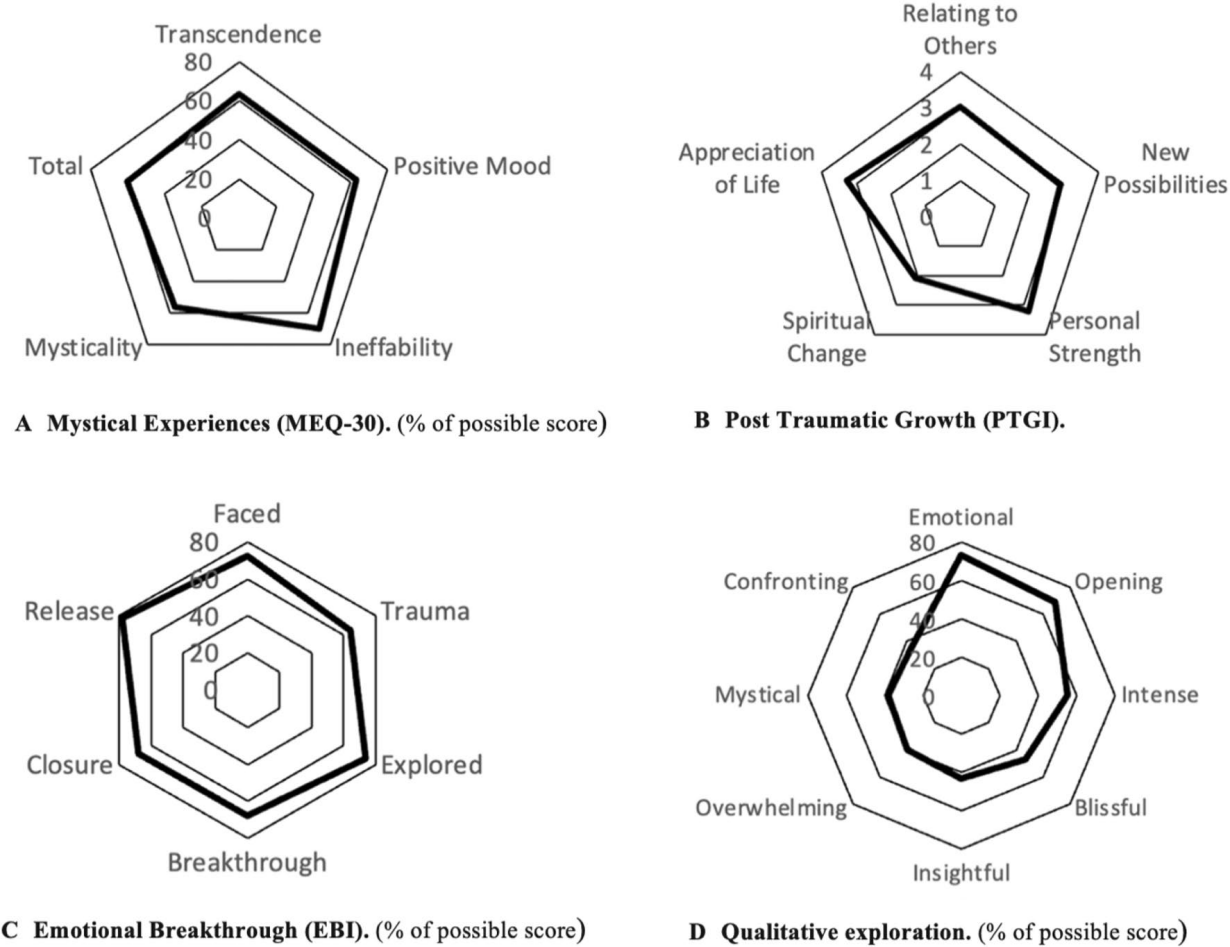
Additional treatment outcomes: mystical experiences, post traumatic growth, emotional breakthrough and qualitative experience

Participants had on average 60% of the total score (90 ± 32.2). This qualifies as ‘*mystical’* (Bouso et al. 2016) and is comparable to a previous study with similar dose (20 mg/70 kg; MEQ_total = 69.56 ± 5.04, *n* = 18; *p* = 0.0613). The factor ‘Ineffability’ was the highest with 70% (10 ± 3.6), followed by ‘Transcendence’ 63% (19 ± 6.4), ‘Positive Mood’ 63% (19 ± 6.8) and ‘Mysticality’ at 56% (42 ± 18.9) (Fig. 3a).

#### PTGI

Participants had an average total score of 63 ± 18.6 out of maximum 105. This is the first study of the PTGI on psilocybin, so no comparison with literature was possible. The two facets with highest scores were ‘Appreciation for life’ (3.3 ± 0.9) and ‘Personal strength (3.2 ± 1.1), followed by ‘Relating to others’ (3.01.1) ‘Seeing new possibilities’ (2.9 ± 0.9) and ‘spiritual change’ (2.1 ± 1.3) (Fig. 3b).

#### EBI

Participants had an average total score of 71 ± 26.3 out of maximum score of 100. This is high compared to an average of 43 ± 31.5 in a sample of 379 participants of an online survey of psilocybin experiences (Roseman et al. 2019) (*p* < 0.0001). The facet ‘emotional release’ was highest (78 ± 27.3), followed by ‘explored challenging emotions and memories’ (74 ± 29.5) ‘faced emotionally difficult feelings’ (73 ± 31.1), ‘closure on an emotional problem.’ (68 ± 31.5), ‘emotional breakthrough’ (68 ± 34.8) and ‘resolution of a personal conflict/trauma’ (64 ± 36.3) (Fig. 3c).

#### Experience description

On a scale from 0 to 100 participants were able to strongly surrender to the experience (82.4 ± 16.0) and felt that it was highly personal significant (85.9 ± 13.4). Further, was the experience rated as emotional (73%) and opening (69%), but also blissful (47%), overwhelming (40%), mystical (38%) and confronting (36%) (Fig. 3d).

### Moderation analysis on changes in personality and psychological complaints over time

**Fig. 4:**
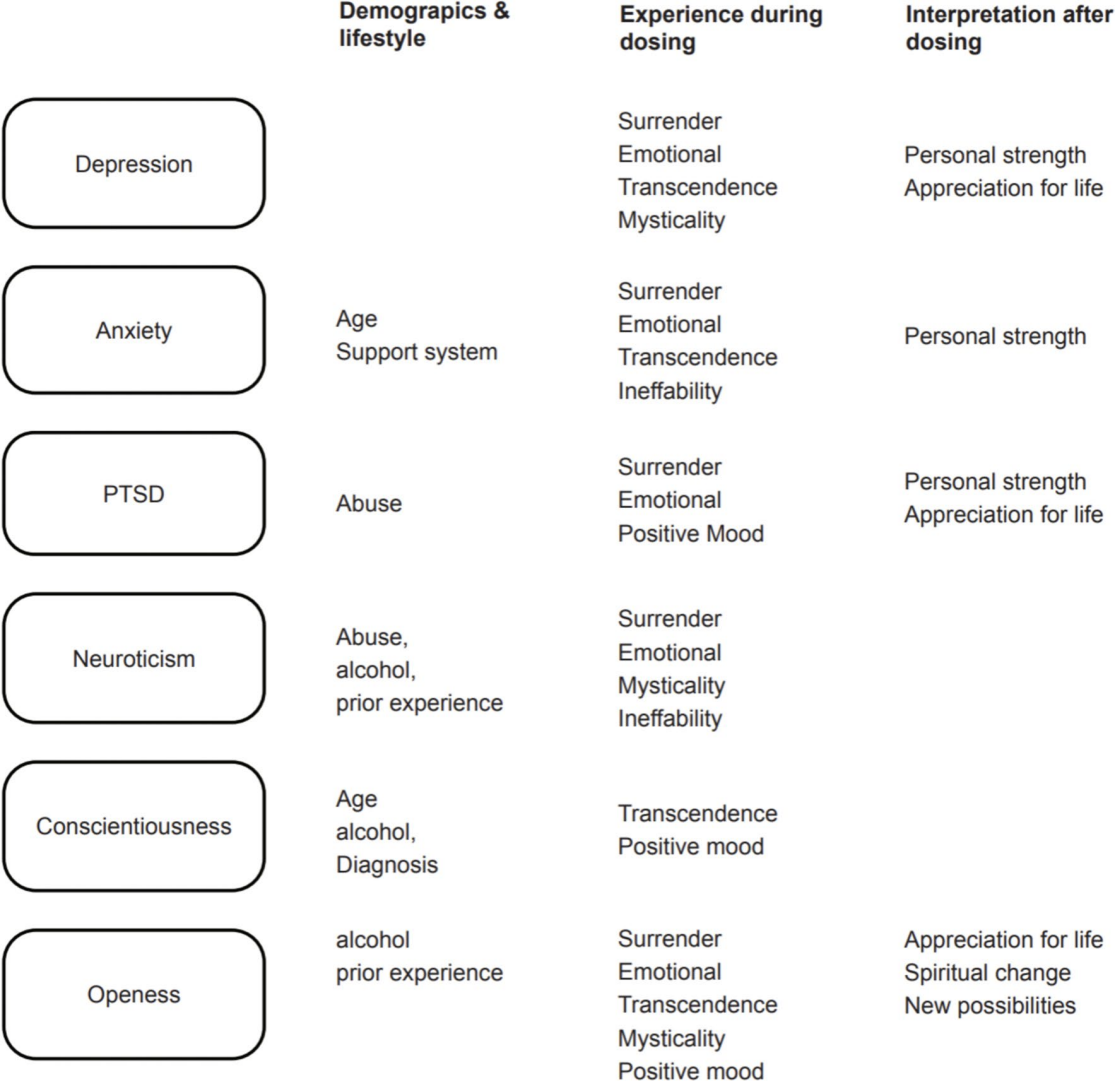
Moderating factors in psilocybin-assisted treatment on mood and personality. Significant factors included demographics & lifestyle, subjective experience during the dosing and personal growth after the program

To check how the demographic background and subjective experience during and after the program were contributing to the observed changes in psychological complaints and personality, the models of the three mood scales (PCL-5, GAD-7, PHQ-9) and the three personality traits (neuroticism, openness and conscientiousness) were extended with moderation variables available from the intake and additional questionnaires (see Table 1 & *description of questionnaires).* In this exploratory analysis, first the influence of demographic moderators present *before* the session were tested, then measures of subjective experience *during* the session, and last interpretations of the experience *1 week after* (see overview of all significant moderators, Fig. 4).

### Influences of demographics & lifestyle moderators before dosing

The available variables were gender, age, alcohol intake, diagnosis, ‘years of having complaints’, reported abuse, support system and ‘prior experience with psilocybin’. Education and employment status were left out of the analysis due to the homogeneity of the participants (see Table 1). Gender and ‘years of having complaints’ did not have an influence on the outcome in any model and were subsequently removed. Including the remaining demographic variables resulted in a valid improvement of the model fit compared to the basic model which only includes the effect of time (anxiety: ∆ AIC = −3.7 depression: ∆ AIC = −5.6 and PTSD: ∆ AIC = −3.82, neuroticism: ∆ AIC = − 46 openness: ∆ AIC = −45.7, conscientiousness ∆ AIC = −67.6,).

**Abuse** showed an interaction with time for PTSD (*F*(2, 39.36) = 5.2 *p* = 0.01) and neuroticism (*F*(2, 33.3) = 4.7, *p* = 0.016). Participants with abuse showed more reduction in symptoms than participants without.

**Professional support system** showed an interaction with time for anxiety (*F*(2, 54.3) = 5.9, *p* = 0.005). Participants with support showed more reduction in symptoms than participants without.

**Alcohol** showed an interaction with time for PTSD (*F*(6, 35) = 4.4, *p* = 0.002) and neuroticism (*F*(6, 35) = 4.4, *p* = 0.002). Participants who drink little alcohol reported the most reduction in symptoms.

**Age** showed an interaction with time for anxiety (*F*(4, 59.6) = 3.3, *p* = 0.016). Younger participant’s anxiety decreased more.

For openness and conscientiousness no interaction with time was tested because of the absence of significant effect over time, as only the pre-post analysis was significant. Main effects for openness were **prior experience** (*F*(4, 31.8) = 3.5, *p* = 0.018) and **alcohol** (*F(*3, 34.1 = 4.4, *p* = 0.010). Participants who had a prior high dose experience and that drink little are in general more open. For conscientiousness main effects are **age** *F*(1 35.5) = 8.0, *p* = 0.008), **alcohol** (*F(*3, 34.2 = 3.9, *p* = 0.017) and **diagnosis** (*F(*3, 34.2 = 3.9, *p* = 0.017). Older participants, participants with a diagnosis and that drink little are more conscientious.

### Subjective experience during dosing moderators

To gain the most comprehensive account of the subjective experience *during* the session from our data we used the subjective reports of degree of ‘Ability to Surrender’ and ‘Emotional experience’ with the 4 facets of the MEQ-30 ‘Mysticality’, ‘Positive Mood’, ‘Transcendence’ and ‘Ineffability’ as possible moderators of the effect. Including subjective experience gave a better model fit then using only time, indicating a better model fit compared to the basic model which only includes the effect of time (depression: ∆ AIC= −384, PTSD: ∆ AIC = −566, anxiety: ∆ AIC = −388, neuroticism: ∆ AIC = −550, openness: ∆ AIC = −435, conscientiousness ∆ AIC = −441).

The ‘**Ability to surrender’** and having an **‘emotional’** experience were moderating the outcomes in all models, except conscientiousness: depression (*F*(1, 28.8) = 11.2, *p* = 0.002)&(*F*(2, 18.5) = 6.5, *p* = 0.007), PTSD (*F*(2, 23.3) = 22.7, *p* < 0.001)&(*F*(2, 26.0) = 11.1, *p* < 0.001), anxiety (*F*(2, 28.6) = 5.0, *p* = 0.033)&(*F*(2, 19.1) = 86.5 *p* < 0.001), neuroticism (*F*(2, 11.9) = 4.4, *p* = 0.038)&(*F*(2, 11.9) = 18.7, *p* < 0.001) and openness (*F*(1, 7.5) = 36.6, *p* < 0.001)&(*F*(1, 7.7) = 27.4, *p* < 0.001).

From the MEQ-30 **‘Transcendence’** was the most important facet, moderating the change in depression (*F*(2, 18.6) = 4.5, *p* = 0.025), anxiety (*F*(2, 22.4) = 11.1, *p* < 0.001), openness (*F*(1, 8.3) = 129.9, *p* < 0.001) and conscientiousness (*F*(1, 11.1) = 36.1, *p* < 0.001). **‘Positive Mood’** moderated the change in PTSD (*F*(2, 26.1) = 4.4, *p* = 0.022), openness (*F*(1, 7.6) = 17.0, *p* = 0.004) and conscientiousness (*F*(1, 12.0) = 12.1, *p* = 0.005). **‘Mysticism’** moderated the change in depression (*F*(2, 18.5) = 8.0, *p* = 0.003), neuroticism (*F*(2, 11.9) = 15.8, *p* < 0.001) and openness (*F*(1, 7.3) = 8.2, *p* = 0.023) and **‘Ineffability’** for anxiety (*F*(2, 18.8) = 19.8, *p* < 0.001) and neuroticism (*F*(2, 11.9) = 12.7, *p* = 0.001).

### Interpretation of growth after dosing

To gain the most comprehensive account of the subjective experience after the session from our data we used the 5 facets of the PTGI: ‘Appreciation for life’, ‘Personal strength’, ‘Relating to others’, ‘Seeing new possibilities’ and ‘Spiritual change’ as possible moderators of the effect. Including these interpretations of growth after the treatment gave a better model fit compared to the basic model which only includes the effect of time (depression: ∆ AIC= −205, PTSD: ∆ AIC = −301, anxiety: ∆ AIC = −208, neuroticism: ∆ AIC = −455, openness: ∆ AIC = −389, conscientiousness ∆ AIC = −395).

**‘Personal strength’** was the most important, moderating the change in PTSD (*F*(1, 46.0) = 4.5, *p* = 0.039), anxiety (*F*(45.3, 1) = 5.0, *p* = 0.031) and depression (*F*(1, 44.1) = 4.8, *p* = 0.034). **‘Appreciation for life’** is moderating the change in PTSD (*F*(1, 45.5) = 4.3, *p* = 0.044), depression (*F*(1, 43.5) = 4.5, *p* = 0.040) and openness (*F*(1, 14.8) = 5.7, *p* = 0.031). Further, openness was also moderated by **‘Seeing new possibilities’** (*F*(, 13.9) = 5.4, *p* = 0.036) and **‘Spiritual change’** (*F*(1, 11.9) = 7.5, *p* = 0.018).

## Discussion

This study focused on moderating factors contributing to changes in the acute and long-term effects of an individual psilocybin-assisted program on depression, anxiety, PTSD and personality structures by including demographic factors, subjective experience and degree of mystical type experiences during the dosing, as well as emotional breakthrough and personal growth after the program.

### Effect on depression, anxiety, PTSD and personality structures

It was found that a single dose of psilocybin in combination with preparation and integration therapy has the potential to lower psychological symptoms of anxiety, depression and PTSD which was maintained at 3 months follow-up. Scores on the personality trait neuroticism also decreased and further went down at 3 months follow-up, while openness and conscientiousness increased only after treatment.

These findings are in line with previous studies (e.g., (Carhart-Harris et al. 2016, 2018; Erritzoe et al. 2018; Griffiths et al. 2016; MacLean et al. 2011) and show that these positive effects could be replicated in an open-label, naturalistic setting. It is important to note, however, that the population in this study exhibited higher scores in depression, PTSD, anxiety and neuroticism than representative samples of the general population.

### MEQ-30, EBI, PTGI

It was found that the individual psilocybin-assisted treatment program induced ‘mystical-type’ experiences, as measured by the Mystical Experience Questionnaire (MEQ-30), which was in a similar range to an earlier study with psylocibin.

Emotional breakthrough, as measured with the Emotional Breakthrough Inventory (EBI), was higher in this study compared to data obtained from other planned psychedelic experiences in non-controlled settings (Roseman et al. 2019). Having a supportive therapeutic environment in this study could have been a factor increasing the emotional safety. Indeed, participants reported that within this set and setting they were able to surrender to the experience to a very high degree, possibly allowing for the emotional breakthrough.

The Post Traumatic Growth Inventory (PTGI) showed a high degree of positive outcome after a single session. This is the first study of the PTGI on psilocybin, therefore results can only be compared to a study with MDMA-assisted psychotherapy, which had a similar outcome measured after 2 sessions (Gorman et al. 2020). Indeed, participants interpreted the experience as highly personally significant.

### Factors influencing positive change in depression, anxiety, PTSD and personality structures

To test which factors of the additional outcomes where important for the positive change in mental health symptoms and personality observed after the program, we conducted an exploratory moderation analysis for the change before, during and after the program.

#### Before

It is noteworthy that gender and duration of participants’ psychological complaints (0 to 10 years) did not have a significant influence on the outcome. The program was effective for relatively acute and for long held complaints and was irrespective of gender.

Abuse was a moderating factor for the changes in neuroticism and in PTSD. Participants who had experienced abuse showed more reduction in symptoms immediately after the session, which continued at the 3-month follow-up. It is promising that participants with abuse show the strongest reduction, considering that this group typically has high baseline scores of neuroticism and PTSD symptomatology. Often, this group shows the least treatment success in traditional approaches, for example in cognitive-behavioral therapy (CBT) (Bagby et al. 2008; Spek et al. 2008; Taylor and Mclean 1993; Wolitzky-Taylor et al. 2012).

High levels of PTSD and neuroticism are associated with rigidity around believes and ruminative cycles impacting an individual’s ability to address the trauma (Ehlers and Clark 2000), while psychedelics actually increase psychological flexibility (Agin-Liebes et al. 2022; Close et al. 2020), also in the presence of depression (Doss et al. 2021; Sloshower et al. 2024) and anxiety (Davis et al. 2020). Further, psychedelics enhanced neural connectivity, particularly in areas involved in emotional regulation and self-referential thought like the default mode network (DMN) (Gattuso et al. 2023) and promote neurogenesis, dendritic growth and synaptic density, which further enhance the brain’s adaptive capabilities and resilience and create a “window of plasticity” that allows for more adaptive thinking patterns and response to therapy (Ly et al. 2018). In line with this motion is also the REBUS (relaxed beliefs under psychedelics) model, suggesting that psychedelics temporarily relax rigid beliefs in the brain’s hierarchical networks, enabling more fluid thinking and emotional openness, both of which are essential for processing and reinterpreting trauma, including abuse (Carhart-Harris and Friston 2019).

For the reduction in anxiety, especially in the long run, it was important to have a personal support system (for example psychologist) outside of this setting. In this study we could show that the benefit of a single psilocybin-assisted session in combination with a continuation of support outside has measurably higher benefits in the long term. This confirms the growing awareness of importance for future treatment plans and policy making to include long-term (integration) support beyond the initial psychedelic session.

It was observed that alcohol intake was moderating the positive effects on neuroticism and openness. Drinking less was associated with more positive outcomes. Alcohol has a known influence on the neurotransmitter systems of dopamine, endogenous opiates, GABA and serotonin (Koob 1996). The 5-HT2A serotonin receptor, which is targeted by psilocybin, is also influenced by alcohol (Belmer et al. 2016), which could give insight why less alcohol use was associated with better outcome.

#### During

The present study also sought to explore how subjective experience during a psychedelic session relates to the observed positive effects. To investigate this, the second analysis included a measure assessing ‘the ability to surrender to the experience’, if the session was ‘perceived as emotional’ and the five facets of the MEQ-30.

The analysis showed that being ‘able to surrender’ to the experience indeed moderated the changes in depression, PTSD, anxiety, neuroticism and openness. Surrendering can be viewed in opposition to rigidity, as discussed above. It allows individuals to let go of conscious control and mental defenses. Reducing this resistance helps accessing deeper states of consciousness where individuals can engage more fully with their emotions (Carhart-Harris and Friston 2019). Surrendering to altered states can also lead to a temporary dissolution of the ego or self-concept (Nour et al. 2016), allowing for new perspectives on identity and personal narratives and ultimately reducing psychological symptoms. Here the role of psilocybin can be seen as a catalyst that supports this letting go into the experience, moving participants through and out of avoidance.

This notion is further supported by the observation that experiencing the session as ‘emotional’ was also an important moderator for the outcome on all the psychological measures. The release of (deep held, or sometimes hard to access) emotions was a major contributor to the changes in the form of reduced experience avoidance (Zeifman et al. 2020). This is reflected in the high degree of emotional breakthrough (71 out of 100) in this study, which might further facilitate the process of fear extinction through exposure and habituation of the fear response. (Nutt and Carhart-Harris 2021; Zeifman et al. 2020).

A previous study using the average scores of the MEQ-30 has shown that mystical-type experience can mediate the effect of psilocybin on therapeutic outcomes (Griffiths et al. 2016). Our analysis was therefore aimed at investigating if there is value in including the individual facets, instead of the total. It was found that indeed different facets moderated different outcome variables.

In our study the facet ‘Transcendence’ (of time, space and boundaries) was the most prevalent and moderated the change in anxiety, depression, openness and conscientiousness. The facet Transcendence describes a sense of timelessness or a departure from the constraints of physical reality during mystical experiences, which can manifest as feelings of unity with the universe or a sense beyond the typical self-concept (or ego dissolution) (Griffiths et al. 2016). This experience has the potential to pull participants beyond existential distress often experienced by participants with severe psychological symptoms, providing the potential of relief (Griffiths et al. 2016).

The observation that different facets of the MEQ-30 were moderating different psychological outcomes indicates that future research focusing on the different aspects of the “mystical experience” could add to the therapeutic process.

#### After

This is the first study to look at a sense of personal growth after the experience by applying the Posttraumatic Growth Questionnaire (PTGI). Therefore, the individual facets of the PTGI were included in a last moderation analysis to explore the possible influence of interpretation after the experience on the outcomes.

The most important facet was **‘**Personal strength’, which was moderating the three psychological complaints PTSD, anxiety and depression. It emphasizes the importance of increased self-reliance felt by the participants after having gone through such an extraordinary experience and is confirming anecdotal feedback in line with “I’ve seen the bottom of it, and I know now that I can handle it” or a “rite of passage”. Such resilience may help navigate future challenges and help confront difficult emotions without becoming overwhelmed, as resilience and increased self-efficacy (believing in the ability to influence one’s own outcomes in the future (Bandura 1997) are associated with better adaptation and coping strategies in the face of trauma (Tugade and Fredrickson 2004). Validating and building on this increased sense of self-reliance showed to be very significant for the integration process.

‘Increased appreciation for life’ was important for the change in PTSD, depression and openness. A renewed appreciation for life can help counteract negative thought loops and cognitive rigidity, which are common in depressive and PTSD patients. This positive shift allows individuals to see value in life experiences, promoting a broader, more optimistic perspective that counters negative beliefs (Carhart-Harris et al. 2018). Additionally, they may feel more motivated to make positive lifestyle changes in the long run resulting from a new zest for life (Griffiths et al. 2016). This points towards the importance of acknowledging, highlighting and building on positive experiences and emotions during, as well as positive interpretations and intentions after the experience for the therapeutic process.

Taken together this study focused on the factors that are supporting immediate and long-term changes in health and personality after an individual psilocybin assisted program. It was possible to replicate positive outcomes from clinical settings in an open-label naturalistic setting and contribute towards highlighting moderating factors useful to consider within the intentional settings for growth and support.

### Limitations

The data was obtained from a naturalistic setting, with participants not enrolled in a clinical study but instead they had searched for and choose this public provider of psychedelic assisted therapy themselves. These participants had the motivation and financial means to enter the program, which makes our sample more naturalistic, but not representative of the general population. There was no comparison of effect of therapy only / psilocybin only, therefore the results can only be taken as a result of the combination of both.

The present results may be partially explained or confounded by the expectations of the participants. As the open-label design does not allow for distinguishing between drug and expectancy effects we tried to get an impression of expectancy by letting therapists assessing participants’ expectations in the intake procedure. Participants reported a variety of positive and negative expectations associated with receiving psilocybin. The variety of expectations illustrated that effects were quite unpredictable for participants, which may be due to their naivety to the use of psilocybin. It must be noted that this assessment only provided a rough measure of conscious expectancy, while subconscious expectancy may be more important, but is inherently unmeasurable (Rucker 2024). The present results can thus partially be explained by expectancy effects. However, we estimate these effects to be a minor factor due to the high dose used in this investigation, and participants reporting a very high degree (82%) of “surrender to the experience”, suggesting a loss of control. This includes loss of possible expectations regarding the treatment. Moreover, the quite large number of dependent variables measured by the questionnaires provides such a detailed registration of changes that global expectations could only minimally have influenced these measures.

## Appendix 1

### Inclusion criteria

Prospective individuals are

- 18 years or older
- Willing to refrain from taking any antidepressants/antipsychotics during the program. This abstinence should commence at least 4 weeks before entering the program. Due to the difficulty and risks associated with tapering off, no participants taking any antidepressants/antipsychotics at the moment of intake were allowed in the program.
- Willing to refrain from taking any benzodiazepines during the 24 h preceding the truffle sclerotia (= 25 mg psilocybin) dosing session.

### Exclusion criteria

Prospective individuals with the following conditions will be excluded:

- Meet DSM-5 criteria for bipolar disorder, schizophrenia, or other psychotic disorders.
- Meet DSM-5 criteria for post-traumatic stress disorder whereby the traumatic event happened less than 5 years ago.
- Meet DSM-5 criteria for dependence on any substance (other than caffeine, nicotine) in the past 30 days.
- Meet DSM-5 criteria for abuse of any substance (other than caffeine, nicotine) in the past 7 days.
- Meet DSM-5 criteria for cluster A or B personality disorders.
- Have first-degree relatives (as parent or full sibling) with past or present psychotic disorders, including schizophrenia, bipolar affective disorder and other psychoses.
- Require concomitant treatment with antipsychotic or antidepressant medications, prescribed for the management of psychiatric symptoms.
- The restriction on 5HT2C/5HT3 antagonists is applicable for 24 h before and including the day of the dosing.
- Have uncontrolled hypertension (bp and hr needs to be assessed during screening).
- Are women who are pregnant or nursing,
- Are reasonably judged to present a serious suicide or homicide risk or who are likely to require psychiatric hospitalization during the course of the program.
- Are unable to fully understand the potential risks and benefits of the treatment and give informed consent.

## Appendix 2

The Psilocybin Program consisted of an intake / screening phase prior to participation in the program. This included a psychiatric and psychological screening for the individuals physical and psychological readiness to participate in the program. All psychiatrists, psychologists, and facilitators were clinically trained mental health professionals. The intake process was designed to exclude participants with a history of psychosis, manic episodes, and / or suicide attempts, first degree relatives with a psychosis or bipolar diagnosis, addiction to psychoactive substances and diagnosis of a cluster A or B personality disorders (schizoid, schizotypal, paranoid, narcissistic, histrionic, antisocial, or borderline).

Before administration of the psychedelic truffle sclerotia (= 25 mg psilocybin) each participant underwent preparatory therapy with both a psychotherapist and a facilitator. The psychotherapy sessions supported each participant with exploring themes, background and emotions that were relevant, so that subconscious material could be brought into the conscious mind and was present for the participant to work through during a dosing session. In addition, preparatory sessions with a facilitator were aimed at building rapport, establishing safety, providing tips and guidance, as well as practical information related to the dosing day.

On dosing sessions, participants ingested the fresh truffle sclerotia at the beginning of the day (09:30am). After ingestion, participants remained in a dosing room with a facilitator (one on one) where they were encouraged to lay on a sofa with a blanket and pillows. Within the room a specific music list was playing in the background and through headphones. Participants were encouraged to focus their attention inwards and were provided with eye masks to support this process. Participants were provided with a healthy, vegetarian meal after cessation of acute effects (5–7 h post-administration). The day following the truffle sclerotia administration, participants discussed their experience with their psychotherapist and had another online session a week after. Each participant received in total 2 h of screening with a psychologist and a psychiatrist and a combined total of 6 h with a psychotherapist and 10 h with a facilitator (including dosing session) throughout the program.

## Appendix 3

The chemical composition of the fresh truffle sclerotia varies from batch to batch. In particular, the production of psychoactive alkaloids may fluctuate substantially. To control for such variability, randomly selected, representative portions of each batch of sclerotia were allocated to chemical analysis. Concentrations of psilocybin and psilocin were determined using high-performance liquid chromatography (HPLC) calibrated with pure reference standards (Sigma-Aldrich). This quantification allowed for the standardization of psilocybin/psilocin doses between batches. Moreover, shelf-life studies were conducted to confirm that concentrations of psilocybin and psilocin within a given batch.
